# Neurodegenerative Disease and Association Football (NDAF): Systematic Review and Meta-Analysis

**DOI:** 10.3390/ijerph22050806

**Published:** 2025-05-21

**Authors:** Nathan E. Howarth, Chen Ji, John Batten, Alan J. Pearce, Helen Dawes, Adam J. White, Gabriele DeLuca, Samantha Bureau, Christopher J. Nowinski, Michelle A. Miller

**Affiliations:** 1 Warwick Medical School, University of Warwick, Coventry CV4 7AL, UK; c.ji.3@warwick.ac.uk (C.J.); adam.white.1@warwick.ac.uk (A.J.W.); michelle.miller@warwick.ac.uk (M.A.M.); 2 NIHR Biomedical Research Centre (BRC), University of Exeter, Exeter EX1 2LU, UK; h.dawes@exeter.ac.uk; 3 Concussion Legacy Foundation, Boston, MA 02115, USA; sbureau@concussionfoundation.org (S.B.); nowinski@concussionfoundation.org (C.J.N.); 4 Faculty of Health and Wellbeing, University of Winchester, Winchester SO22 4NR, UK; john.batten@winchester.ac.uk; 5 Swinburne Neuroimaging Facility, School of Health Science, Swinburne University, Melbourne 3122, Australia; apearce1@swin.edu.au; 6 Professional Footballers Association, London EC3M 8AA, UK; 7 NIHR Biomedical Research Centre (BRC), University of Oxford, Oxford OX3 9DU, UK; gabriele.deluca@ndcn.ox.ac.uk

**Keywords:** concussion, head impact, neurodegeneration, association football

## Abstract

There is increasing concern that head injuries in Association Football (or soccer) may lead to adverse health outcomes. The aim of this study was to determine whether head impacts or injuries are associated with an increased risk of neurodegenerative disease. We performed a systematic search using PubMed, Embase, and Ovid (up to April 2025). Studies included investigated neurodegenerative diseases in football in comparison to control athletic and general populations. Data were extracted according to PRISMA guidelines. Studies with an odds ratio (OR) were included in the meta-analysis. A total of ten studies were included in this review, of which nine were suitable for meta-analysis from eight cohorts. The risk for developing any neurodegeneration was 1.69 OR (95%CI 1.11 to 2.59; *p* = 0.01); for Dementia, it was 2.16 OR (95%CI 1.60 to 2.93; *p* < 0.01; for Motor Neurone Disease (MND), it was 1.39 OR (95%CI 0.67 to 2.53; *p* = 0.21); for Parkinson’s Disease (PD), it was 1.14 OR (95%CI 0.55 to 2.89; *p* = 0.79). Heterogeneity was reduced following the removal of two studies and the revised risk scores for any neurodegenerative disease; Dementia increased, with that for MND reaching significance, 1.81 OR (95%CI 1.22 to 2.30; *p* = 0.01), but there remained no association with PD. Evidence suggests that professional football significantly increases the odds of neurodegenerative disease.

## 1. Introduction

Association Football, or soccer, (herein football) has seen, like other contact or collision sports, concern regarding head impact exposure and the development of neurodegenerative disease. During the early 2000s, concern was raised in Italy about the potential increase in Amyotrophic Lateral Sclerosis (ALS) in ex-professional footballers [1,2]. Concurrent concerns were raised by the Astle Family about the death of Jeff Astle from a neurodegenerative disease, which was later diagnosed as the first case of Chronic Traumatic Encephalopathy (CTE) in football in the UK [3]. Since then, a marked increase in CTE amongst former contact sport players has been reported, including in the first FIELD study, which reported that ex-footballers in Scotland had a five times greater risk of neurodegenerative disease [4].

Football has been associated with neurological symptoms during life and neuropathological evidence after death of CTE [5,6], Parkinson’s Disease (PD) [7], ALS, and Motor Neurone Disease (MND; herein ALS will be MND) [7], Alzheimer’s Disease (AD), and Dementia [7,8,9,10]. Whilst the reasons for this remain to be fully clarified, it has been shown that repetitive head impacts (RHI) in football, such as heading the ball, as well as other traumatic brain injury (TBI) may lead to the development of Chronic Traumatic Encephalopathy (CTE) [6] and other neurological conditions. These injuries have also been associated with Alzheimer’s Disease (AD) and Dementia [7,8,9], Parkinson’s Disease (PD) [7,9], and MND [7,9]. These studies, however, are often limited by their observational design, and there is a need to conduct follow-up studies in both male and female professional sports personnel and to address such risks in younger non-professional players [11]. Understanding the mechanistic pathways is also important. Evidence suggests that, during a head impact, the accelerative forces generated may cause brain tissue injury and the release of different structural proteins into different fluids within the body, including neurofilament light chain. It is possible that these proteins might be useful biomarkers for future studies [12]. Given the emerging relationship between the brain and future neurological health, it is important to determine the strength of the relationship and possible causal pathways so that better screening programs for these conditions may be developed and future generations of sports personnel may be protected. This would have the potential to improve both physical and mental health outcomes in sports personnel.

The aim of this study was to undertake a systematic review and meta-analysis of neurodegeneration in former footballers.

## 2. Methods

### 2.1. Search Strategy

The search (designed by NEH, MAM, AJP, and JB) was undertaken across three platforms (PubMed, OVID, and EMBASE). The search terms for this review were the following, Football: Association Football, Soccer, and Neurodegenerative Disease: Chronic Traumatic Encephalopathy, CTE, Alzheimer’s, MND, Motor Neurone Disease, ALS, Amyotrophic Lateral Sclerosis, Dementia, Vascular Dementia, and Parkinson’s Disease. Searches occurred up till 28 April 2025.

### 2.2. Title and Abstract Analysis

The blinded title and abstract were first screened by two authors (NEH and AJW) to determine which papers were suitable for inclusion in full text, and a third author was available for arbitration but was not required.

### 2.3. Inclusion and Exclusion Criteria

Studies were included for full-text analysis if the studies were full text, cohort studies, and had a population of football players.

Studies excluded were those that only had a meeting abstract or unpublished material available or where data were not available for analysis.

### 2.4. Full-Text Analysis

Full-text extraction was followed by blind analysis for inclusion in meta-analysis and review (by NEH and AJW; a third author was available for arbitration but was not required); an odds ratio either needed to be provided or possible to calculate by a cohort compared to controls. In cohort studies that had more than one study, the most recent from the cohort was included. If there was a study where the odds ratio for footballers was indeterminable, then the authors were contacted for the dataset, with the study being removed if no data were forthcoming. After full-text analysis, studies meeting the criteria were extracted for quality assessment and data extraction.

### 2.5. Quality Assessment

Quality assessment was undertaken independently by two authors (NEH and HD) using the Joanna Briggs Institute (JBI) checklist for case-control studies and the JBI checklist for cohort studies as appropriate. A third author (MAM) arbitrated the scores with any discrepancies.

### 2.6. Meta-Analysis

The studies included were either cohort studies in which footballers’ rates of neurodegenerative diseases were compared with controls or a cohort with neurodegenerative diseases that had their risk factors compared across the cohort and their controls.

Odds ratios (ORs) were calculated in the cohorts for both footballers and controls based on having or not having neurodegenerative disease. The ORs for neurodegenerative disease either encompassed all neurodegenerative diseases or were subdivided into different neurodegenerative diseases (Alzheimer’s, Early Onset Dementia (EOD), Early Onset Frontal Dementia (EOFD), and Motor Neurone Disease (MND)). If multiple published reports from the same study were available, for each outcome of interest, we included only the one with the most detailed information for both exposure and outcome.

In neurodegenerative disease cohorts, the OR was produced based upon the calculation of the footballers with neurodegenerative disease in their cohort and the footballers in the control without. The meta-analysis of the ORs was undertaken within STATA 18.0 2025 using the Radon-effects REML model.

The influence of individual studies, from which the meta-analysis estimates are derived, was examined by omitting one study at a time to determine the extent to which inferences depend on each study or group of studies (sensitivity analysis).

Sub-group analyses were also carried out to examine sources of heterogeneity that may be attributable to characteristics of the study or study design.

### 2.7. Publication Bias

We assessed the risk of publication bias by using a funnel plot to assess the asymmetry of publications and by performing Egger’s regression test.

## 3. Results

**Figure 1:**
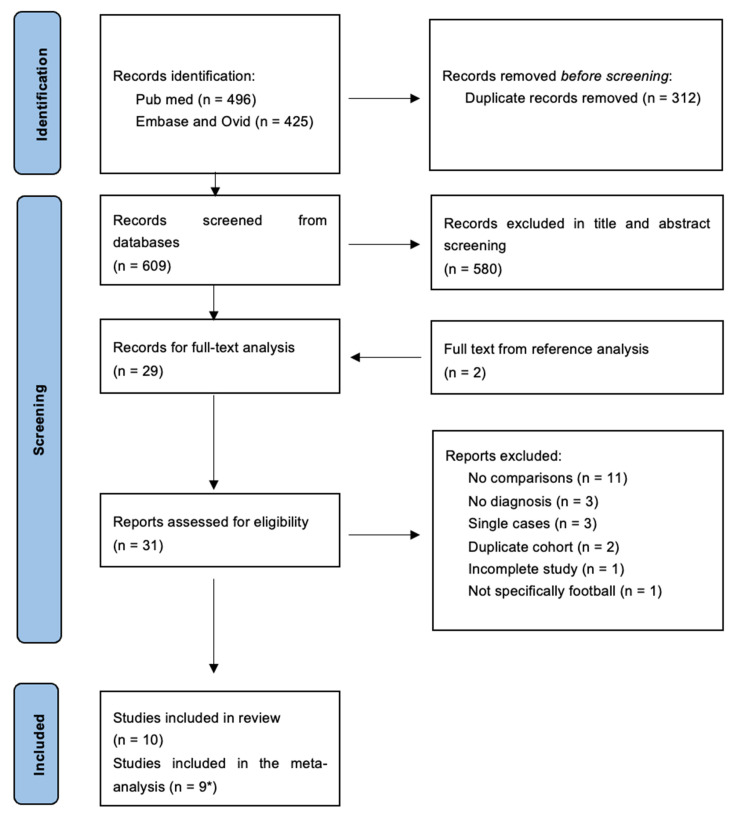
Prisma flow chart. * Nine studies were included from eight cohorts. Russell [7] had data available for overall neurodegeneration, Parkinson’s Disease, and MND. Russell [8] was an updated study from the FIELD Study Cohort focusing on Dementia and presented more recent Dementia data, and this study was included in the Dementia meta-analysis.

A total of 921 studies yielded 609 after duplicates were removed (see Figure 1). The title and abstract analysis gave 29 studies for full-text analysis, with a further two identified from reference lists. Of these studies, one did not differentiate contact sports being practised (authors were contacted without a response); one study was not a full study; two studies were removed as duplicate cohorts (Chio et al. [13] is a duplicate of Chio et al. [14], and Mackay et al. [4] is a duplicate of Russell et al. [7]); three had no diagnosis; three were single cases; eleven studies were without a comparison group. Therefore, a total of ten studies were included [7,8,9,10,14,15,16,17,18,19], of which nine were suitable for meta-analysis from eight cohorts; one study did not produce an OR specific to football. For the FIELD study, updated information was available [8], but only for the Dementia outcome. The earlier FIELD study [7] has, therefore, been included for neurodegenerative disease, PD, and MND. Table 1 represents the overall study populations and characteristics of the studies.

### 3.1. Population

The nine included cohorts have a total population of 108,631. The population includes 22,322 exposed to football and 86,309 non-exposed. Across the cohorts 5405 had neurodegenerative disease, 1084 were exposed to football, and 4321 were not exposed to football. In the meta-analysis the eight included studies had a population of 97,632, with 14,997 exposed to football and 82,635 non-exposed, with 5397 had neurodegenerative disease, 1076 were exposed to football, and 4321 not exposed to football. 

### 3.2. Neurodegenerative Diseases, Dementia

Five studies reported the rates of Dementia. One study differentiated AD from Dementia, and Batty et al. [16] reported an age-adjusted hazard ratio (HR) of 1.80 (95% CI 1.27 to 2.54) for Dementia. Three studies did not differentiate Dementia and AD; Macnab et al. [10] reported an OR of 2.92, Russell et al. [8] reported an HR of 3.02 (95% CI 2.54 to 3.58), and Ueda et al. [9] reported an HR of 1.62 (95% CI 1.47 to 1.78). One study, Adani [15], looked at differentiated Early Onset Dementias with an overall OR of 1.72 (95% CI 0.54 to 9.26).

### 3.3. Alzheimer’s Disease (AD)

One study differentiated the diagnosis of Dementia and AD. Batty et al. [16] reported an age-adjusted HR of 2.43 95% CI 1.62 to 3.64).

### 3.4. Early Onset Dementias

One study, Adani et al. [15], reported rates of Early Onset Frontal-Temporal (EOFTD) Dementia and Early Onset Alzheimer’s Disease (EOAD) (OR for EOFTD as 2.34 (95% CI 0.43 to 15.90) and an OR for EOAD as 1.29 (95% CI 0.32 to 10.06).

### 3.5. Motor Neurone Disease (MND)

Six studies reported on MND; Chen et al. [17] reported an OR of 1.43 (95% CI 0.95 to 2.06), Chio et al. [14] reported a standardised mortality rate of 6.45 (95% CI 2.78 to 12.70), Filippini et al. [18] reported an OR of 0.94 for all footballers and 1.45 for professional footballers, Russell et al. [7] reported an HR of 3.52 (95% CI 1.81 to 6.88), and Ueda et al. [9] reported an HR of 1.27 (95% CI 0.73 to 2.22).

### 3.6. Parkinson’s Disease (PD)

Two studies reported on PD; Russell et al. [7] reported an HR of 2.09 (95% CI 1.20 to 3.61), and Ueda et al. [9] reported an HR for PD of 0.70 (95% CI 0.48 to 1.03).

### 3.7. Location

Across the ten studies, there were nine European studies and one non-European study. Of the European studies, four were from Italy [2,14,15,18], two were from the UK (two based in Scotland [7,8], from the same cohort, and one in the East Midlands [10]), one was from Finland [16], and one was from Sweden [9]. The study from outside of Europe was from New Zealand [17].

### 3.8. Study Designs

Four cohorts were cohort studies [7,9,14,16], four cohorts were case-control studies [2,15,17,18], and one cohorts was a cross-sectional study [10].

### 3.9. Diagnosis

One study used self-reported diagnosis (Macnab et al. [10]), with the other studies using either direct diagnosis in clinics or data from medical records.

### 3.10. Quality Assessment

The results of the quality assessment using JBI are presented in Table 2. Arbitration was required for two papers [2,15].

### 3.11. Meta-Analysis

The meta-analysis focused on the OR for neurodegenerative disease. One paper did not meet the inclusion for meta-analysis as they could not produce an OR. This was because they did not observe any MND cases in their control population (Chio et al. [14]). Therefore, nine studies from eight cohorts were included in the meta-analysis.

### 3.12. Meta-Analysis: All Neurodegenerative Disease

**Figure 2:**
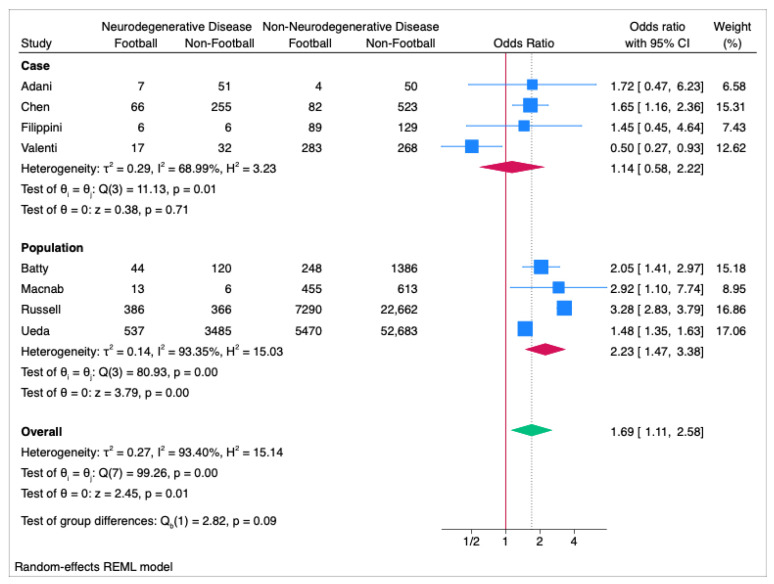
Forest Plot of all neurodegenerative disease ORs across the studies, split into subset analysis of case and population (cohort and cross-sectional) studies.

The results of the meta-analysis across all neurodegenerative conditions for the eight included cohorts were 1.69 OR (95%CI 1.11 to 2.58; *p* = 0.01 (see Figure 2) with significant heterogeneity noted between them (I^2^ = 93.40%; *p* < 0.01). Subset analysis based on research design revealed that case-control studies were not significant, 1.14 OR (95%CI 0.58 to 2.22; *p* = 0.71), but demonstrated significant heterogeneity (I^2^ of 68.99%; *p* = 0.01). Population cohort and cross-sectional studies were significant, with an OR of 2.23 (95%CI 1.47 to 3.38; *p* < 0.01) with significant heterogeneity (I^2^ = 93.35%; *p* < 0.01).

### 3.13. Meta-Analysis: Dementia

**Figure 3:**
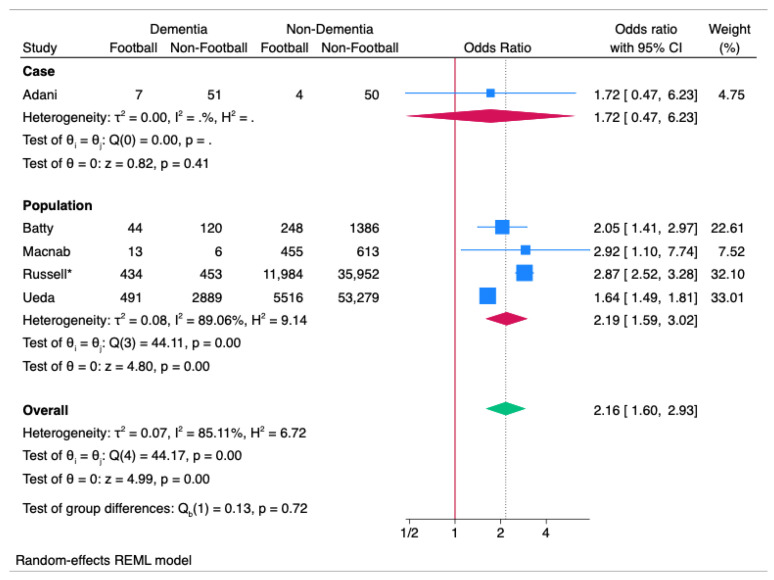
Forest Plot of Dementia ORs across the studies, split into subset analysis of case and population (cohort and cross-sectional) studies. * Russell [8] has replaced Russell [7] as the latest study from the FIELD Study Cohort with the most recent Dementia data.

Meta-analysis of the Dementia studies demonstrated an overall OR of 2.16 (95%CI 1.60 to 2.93; *p* < 0.01) (see Figure 3) with significant heterogeneity (I^2^ = 85.11%; *p* < 0.01). There was only one case-control study that was not significant. Population-based studies were significant, with 2.19 OR (95%CI 1.59 to 3.02; *p* < 0.01) with heterogeneity (I^2^ = 89.06%; *p* < 0.01).

### 3.14. Meta-Analysis: MND

**Figure 4:**
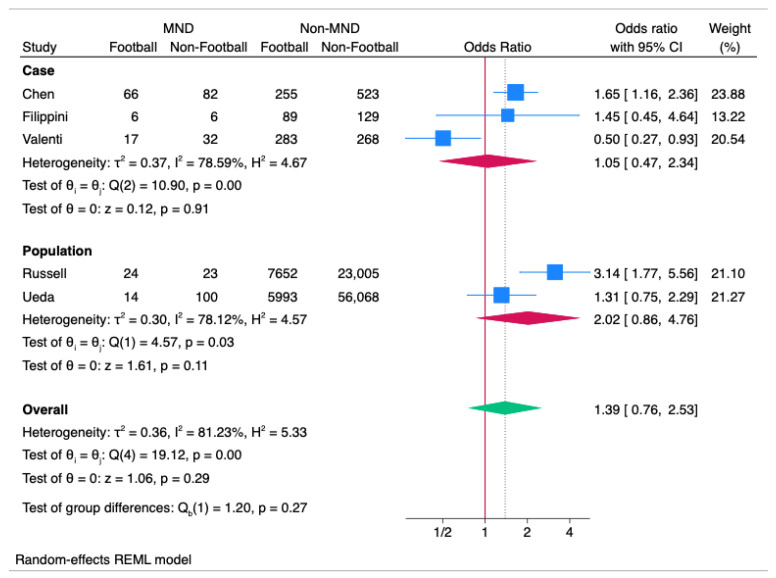
Forest Plot of MND ORs across the studies, split into subset analysis of case and population (cohort and cross-sectional) studies.

Meta-analysis of the MND studies demonstrated a non-significant overall effect of 1.39 OR (95%CI 0.76 to 2.53; *p* = 0.21) with significant heterogeneity (I^2^ = 81.23%; *p* < 0.01 (see Figure 4). Both case-control and population-based studies had non-significant effects (case-control studies: 1.05 OR (95%CI 0.47 to 2.34; *p* = 0.91) with significant heterogeneity (I^2^ = 78.59%; *p* < 0.01); population-based studies: 2.02 OR (95%CI 0.86 to 4.76; *p* = 0.11) with significant heterogeneity (I^2^ = 78.12%; *p* = 0.03)).

### 3.15. Meta-Analysis: PD

**Figure 5:**
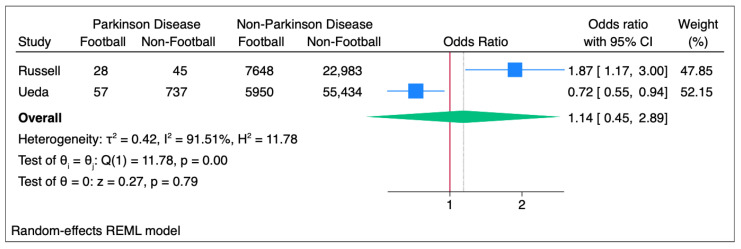
Forest Plot of PD ORs across the studies, split into subset analysis of case and population (cohort and cross-sectional) studies.

Meta-analysis of the subset of PD ORs demonstrated a non-significant overall effect of 1.14 OR (95%CI 0.45 to 2.89; *p* = 0.79) with significant heterogeneity (I^2^ = 91.51%; *p* < 0.01 (see Figure 5). Both studies with analysis of PD were population-based studies.

### 3.16. Sensitivity Analysis: All Neurodegenerative Disease

Total group: Stepwise removal of each study had no major effects on outcome except for the removal of Valenti et al. [2]. Removal of this study reduced the heterogeneity, although this was still significant (I^2^ = 85.76%; *p* < 0.01), but resulted in an increase in the effect size to 2.03 OR (95%CI 1.49 to 2.76; *p* = 0.01).

Sub-group case-controls: Removal of Valenti et al. [2] resulted in a marked decrease in heterogeneity to a non-significant level (I^2^ = 0.00%; *p* = 0.98), with the overall effect now significant with an OR of 1.64 (95%CI 1.18 to 2.28; *p* < 0.01).

Sub-group population studies: Removal of Ueda et al. [9] resulted in non-significant heterogeneity (I^2^ = 62.49%; *p* = 0.07) and increased the effect size to 2.25 OR (95%CI 1.62 to 3.13; *p* = 0.01).

### 3.17. Sensitivity Analysis: Dementia

Total group: Removal of Ueda et al. [9] resulted in non-significant heterogeneity (I^2^ = 32.96%; *p* = 0.34) and increased the overall effect to an OR of 2.56 (95%CI 1.97 to 3.31; *p* < 0.01).

Removal of Russell et al. [8] resulted in non-significant heterogeneity (I^2^ = 17.09%; *p* = 0.47) but decreased the overall effect size to 1.75 OR (95%CI 1.45 to 2.11; *p* < 0.01).

Sub-group population cohorts: Removal of Ueda et al. [9] resulted in non-significant heterogeneity (I^2^ = 41.03%; *p* = 0.24) and increased the effect to an OR of 2.60 (95%CI 1.99 to 3.39; *p* < 0.01). Removal of Russell et al. [8] resulted in non-significant heterogeneity (I^2^ = 27.19%; *p* = 0.28) but decreased the overall effect size to an OR of 1.77 (95%CI 1.44 to 2.17; *p* < 0.01).

### 3.18. MND

Total group: The removal of Valenti et al. [2] resulted in non-significant heterogeneity (I^2^ = 46.82%; *p* = 0.16), with the overall effect becoming significant with an OR of 1.81 (95%CI 1.22 to 2.30; *p* = 0.01).

Case-control studies: Removal of Valenti et al. [2] reduced the heterogeneity to non-significant I^2^ = 0.00%; *p* = 0.83), with the effect becoming significant with 1.63 OR (95%CI 1.16 to 2.30; *p* < 0.01).

### 3.19. Publication Bias

**Figure 6:**
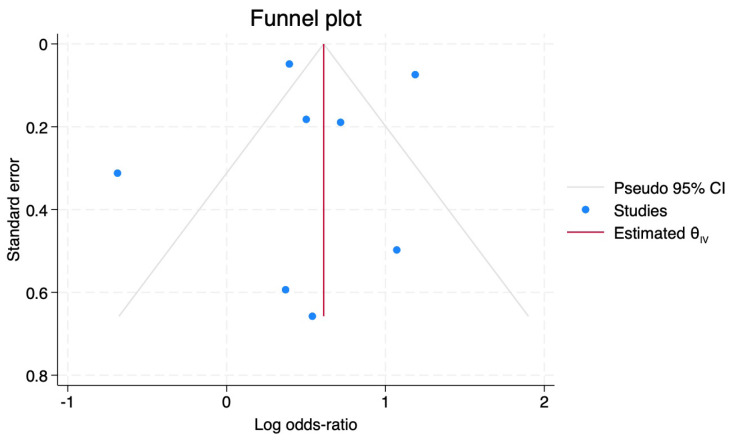
Funnel Plot of the publications included in the meta-analysis.

The Egger’s regression test demonstrated no small study effect and a low risk of publication bias (*p* = 0.7006). The funnel plot also demonstrated symmetry between publications (see Figure 6).

## 4. Discussion

A total of ten studies were included in this review, of which nine were suitable for meta-analysis from eight cohorts. The results, which demonstrated low publication bias, provide evidence that the risk of neurodegenerative disease is increased in footballers compared to control populations with an increased OR of 69% for developing any neurodegeneration, 37% for MND, and 116% for Dementia. However, considerable heterogeneity between studies was observed, and a significant effect (81%) was seen for MND when the heterogeneity was reduced by the removal of two studies, but there remained no association with PD. The variability within the findings and the observed variability seen within individual studies suggests that the risk of developing neurodegenerative disease within footballers may be disease-dependent and raises caution on the generalisability of the findings.

Case-control studies have a lower OR rate across all neurodegenerative diseases with fewer cases and controls compared to the included cross-sectional and cohort studies. Further methodological concerns exist in the meta-analysis with the case cohorts. In Adani et al. [15], the controls were recruited as the caregivers of those with Dementia, which could introduce a bias due to environmental risk factors in the home. Additionally, the study by Valentia et al. [2], an early study on MND, is of poor quality due to the study design. Chen et al. [17] aimed to recruit two controls per case; although age and gender-matched, the age of the cases was mostly under 60 years and mostly over 70 years in the controls. Over-representation in cases over controls and being younger strengthens the interpretation that football exposure is a relevant factor for neurodegenerative disease risk.

In the cohort studies, it is expected that studies should aim to recruit large numbers of controls. Of the three cohort studies, two recruited ten controls to one case [7,9]. In Batty et al. [16], the control group was recruited from military and civic service.

Further to Ueda et al. [9] this study had a mixture of professional and non-professionals, giving the spread of studies, mainly in case-control studies. Fillipini et al. [18] allowed for the differentiation of competitive football, which was included in this study’s analysis, but it is unclear if this means professional football.

The meta-analysis across all the sub-group analyses demonstrated significant heterogeneity and, therefore, a spread of effect. Subsequent sensitivity analysis has highlighted potential outliers in the data. The funnel plot has also demonstrated there are studies outside of expected parameters, although Egger’s test is non-significant. Valenti et al. [2] was one of the original papers on this topic, and, although it is included in this dataset through meeting the inclusion criteria, it has some quality and design challenges (see Table 2). Within the design, it is not clear what is defined as competitive football. Valenti’s [2] study was an outlier in terms of the quality of the dataset, both Russell [7] and Ueda [9] used large population datasets with small standard errors.

The study by Ueda et al. [9] is a large cohort study but includes both professional and non-professional individuals. The heterogeneity was no longer significant after the removal of this study, and there was an increase in the observed effect size for all neurodegenerative diseases, Dementia, and MND. A further explanation could also come from the frequency of games. In France, ex-professional footballer Dementia morality produced an SMR of 3.38 [19]. This rate of mortality for Dementia is like that of Russell et al. [8]’s HR of 3.02. For Ueda [9], the mortality was HR 1.69. A crude comparison of the frequency of games demonstrates a difference between Scotland, France, and Sweden. In 1970, Celtic played 66 games [20]; they played 42 games in the French topflight in 1970 Olympique de Marseille [21], and they played 30 games in Sweden Malmo during 1970 [22]. Any comparison could also be influenced by squad size and training.

In this systematic review, studies report in a varying manner with HRs, SMRs, and ORs. Therefore, this study is limited to crude and unadjusted ORs from within the studies. The use of ORs as the measure in the meta-analysis also removed Chio et al. [14] from the meta-analysis; despite having a control cohort, no cases of MND were reported, so no OR was produced. Additionally, this study’s design includes studies with control cohorts instead of expected rates of neurodegeneration, and this excludes Orhant and colleagues [19]. In their study, Orhant et al. [19] used SMRs and did not have a control group, although they had a similar mortality to Russell et al. [7].

### Future Research

More research is required into the rates and risk of neurodegeneration in football. Larger cohorts are required to investigate all neurodegenerative diseases, and additional focus on rarer neurological diseases, such as MND, is required. Any future studies should attend to their design to optimise age- and gender-matching and representation in studies. Further representations in future studies could consider socio-economic backgrounds. Studies might also seek to include CTE or those suspected of having CTE (post-mortem) or traumatic encephalopathy syndrome (during life), now that there is increasing knowledge and awareness of the condition. Investigations should also determine the benefits and differences between using general populations and athletic populations as a control population.

## 5. Conclusions

This systematic review and its results from the meta-analyses have demonstrated that there is a significantly increased risk of neurodegenerative disease in ex-football players, although no significant association with PD was observed. The sensitivity analysis demonstrated that, when the degree of heterogeneity between studies is reduced, the associated risk is significantly increased. Further research with good quality cohort studies is required to explore these findings in more detail and to determine whether any interventions may mitigate this effect and improve both past and current players’ existing and future health.

## Figures and Tables

**Table 1:** The characteristics of included studies.

Study	Study Characteristics	Population
Adani [15]	Italian study specifically looking at Early Onset Dementia (EOD). Mixture of former amateurs and professional footballers. The matched controls to EOD were the caregivers irrespective of age.	Total 112 Football Dementia 7, Non-Football Dementia 51, Football Non-Dementia 4, Non-Football Non-Dementia 50
Batty [16]	Finnish study with surveillance ending in 2015. Mixture of former amateurs and professional footballers. Controls from military and civil servants were age-, gender-, and socio-economically matched.	Total 1798 Football Dementia 44, Non-Football Dementia 120, Football Non-Dementia 248, Non-Football Non-Dementia 1386
Chen [17]	New Zealand MND nationwide surveillance 2013–2016. Mixture of former amateurs and professional footballers. Controls from general population, which were socio-economically matched. The general population had an over-representation of individuals in their 70s.	Total 926 Football MND 66, Non-Football MND 255, Football Non-MND 82, Non-Football Non-MND 523
Chio * [14]	Italian study focused on MND cases in former professional footballers. Controls are athletic populations, cyclists and basketballers. Between 1970 and 2006.	Total 10,999 Football MND 8, Non-Football MND 0, Football Non-MND 7317, Non-Football Non-MND 3674
Filippini [18]	Italian study on MND 2008 to 2011 in two regions and 2002–2012 to a third. Cases of competitive football were recruited through discharge letters or death certificates. Controls were recruited from the general population who was age-, sex-, and socio-economically matched.	Total 230 Football MND 6, Non-Football MND 6, Football Non-MND 89, Non-Football Non-MND 129
Macnab [10]	Cross-sectional study into Dementia in Nottinghamshire, UK. Self-disclosed diagnosis in both the former professional footballers and age- and sex-matched general population controls.	Total 1087 Football Dementia 13, Non-Football Dementia 6, Non-Football Non-Dementia 455, Non-Football Non-Dementia 613
Russell ** [7] Included for Neurodegenerative Disease, Parkinson’s Disease, and MND analysis.	Scottish cohort study following former professional footballers with age- and gender-matched general population controls for Alzheimer’s, MND, and Parkinson’s Disease. Based upon clinical records through prescriptions and death certificates. Collection ended December 2018.	Total 30,704 Football Neurodegeneration 386, Non-Football Neurodegeneration 366, Football Non-Neurodegeneration 7290, Non-Football Non-Neurodegeneration 22,662
Russell ** [8] Included for Dementia analysis.	Scottish cohort study following former professionals with age- and gender-matched general population controls for Alzheimer’s, MND, and Parkinson’s Disease. Based upon clinical records through prescriptions and death certificates. Collection ended in December 2021.	Total 47,936 Football Dementia 434, Non-Football Dementia 453, Football Non-Dementia 11,550, Non-Football Non-Dementia 35,499
Ueda [9]	Swedish cohort study of amateur and professional based upon prescription data and death certificates. Controls were matched based on year of birth, locality, and sex.	Total 62,175 Football Neurodegeneration 537, Non-Football Neurodegeneration 3485, Football Non-Neurodegeneration 5470, Non-Football Non-Neurodegeneration 52,683
Valenti [2]	Italian MND study from January 2002 to May 2003 with competitive and amateur football practice in Neurology clinics and control populations from general population locally, age- and sex-matched.	Total 600 Football MND 17, Non-Football MND 32, Football Non-MND 283, Non-Football Non-MND 268

* The study by Chio et al. [14] reported a standardised mortality rate but not OR and was not included in the meta-analysis. ** Two studies report on the same cohort. We selected the study with the most detailed and updated data for each outcome.

**Table 2:** JBI quality assessment for studies included in this review. y = yes, n = n, na = not applicable.

JBI Case-Control Studies	Adani [15]	Chen [17]	Filippini [18]	Valenti [2]	JBI Cohort Studies	Batty [16]	Chio [14]	Russell [7]	Russell [8]	Ueda [9]	JBI Cross-Sectional Studies	Macnab [10]
**1. Were the groups comparable other than the presence of disease in cases or the absence of disease in controls?**	n	y	Y	y	**1. Were the two groups similar and recruited from the same population?**	n	n	n	n	y	**1. Were the criteria for inclusion in the sample clearly defined?**	y
**2. Were cases and controls matched appropriately?**	n	n	Y	y	**2. Were the exposures measured similarly to assign people to both exposed and unexposed groups?**	y	y	y	y	y	**2. Were the study subjects and the setting described in detail?**	y
**3. Were the same criteria used for identification of cases and controls?**	n	y	y	y	**3. Was the exposure measured in a valid and reliable way?**	y	y	y	y	y	**3. Was the exposure measured in a valid and reliable way?**	n
**4. Was exposure measured in a standard, valid, and reliable way?**	u	y	y	y	**4. Were confounding factors identified?**	y	n	y	y	y	**4. Were objective, standard criteria used for measurement of the condition?**	n
**5. Was exposure measured in the same way for cases and controls?**	y	y	y	u	**5. Were strategies to deal with confounding factors stated?**	u	u	y	y	y	**5. Were confounding factors identified?**	y
**6. Were confounding factors identified?**	y	y	y	y	**6. Were the groups/participants free of the outcome at the start of the study (or at the moment of exposure)?**	u	u	u	u	u	**6. Were strategies to deal with confounding factors stated?**	y
**7. Were strategies to deal with confounding factors stated?**	u	y	y	y	**7. Were the outcomes measured in a valid and reliable way?**	y	y	y	y	y	**7. Were the outcomes measured in a valid and reliable way?**	y
**8. Were outcomes assessed in a standard, valid, and reliable way for cases and controls?**	y	y	y	u	**8. Was the follow**-**up time reported and sufficient to be long enough for outcomes to occur?**	y	y	y	y	y	**8. Was appropriate statistical analysis used?**	y
**9. Was the exposure period of interest long enough to be meaningful?**	u	u	u	u	**9. Was follow up complete, and if not, were the reasons for loss to follow up described and explored?**	y	n	y	y	y		
**10. Was appropriate statistical analysis used?**	u	y	y	y	**10. Were strategies to address incomplete follow up utilized?**	na	y	na	na	na		
					**11. Was appropriate statistical analysis used?**	y	y	y	y	y		
